# Lack of evidence of hepatitis in patients with oral lichen planus in China: A case control study

**DOI:** 10.4317/medoral.20812

**Published:** 2016-01-31

**Authors:** Jiangyuan Song, Zhihui Zhang, Xiaoli Ji, Sha Su, Xiaodan Liu, Si Xu, Ying Han, Dongdong Mu, Hongwei Liu

**Affiliations:** 1 MD. PhD. PhD, MD. Post graduate. Department of Oral Medicine, Peking University School and Hospital of Stomatology, 22 South Zhong Guan Cun Street, Beijing 100081, China; 2 PhD. Stomatology Department, Peking University Third Hospital, Beijing, China

## Abstract

**Background:**

China has been one of the countries with high prevalence of chronic hepatitis B virus (HBV) and hepatitis C virus (HCV) liver disease. And lichen planus is an extrahepatic manifestation of patients with chronic HCV infection. This case-control study was conducted to investigate the relationship between oral lichen planus (OLP) and HBV/HCV infection in China.

**Material and Methods:**

A total of 776 patients, including 150 patients with OLP (Group OLP), 429 inpatients from the Trauma Ward of Oral and Maxillofacial Surgery Department (Group A), 110 patients with other oral mucosal diseases, but without a reported association with HCV infection (Group B) and 87 patients with oral lichenoid lesion (Group OLL), were compared with their seroprevalence of anti-HCV antibody (HCVAb), hepatitis B surface antigen (HBsAg) and the parameters of liver functions. Moreover, the clinical characteristics of OLP were also observed, such as gender, age, chief complaint, course of the disease, clinical type, sites involved and so on.

**Results:**

The positive rates of HCVAb and HBsAg in OLP patients were 0.7% and 4%, respectively. Neither HCVAb nor HBsAg was associated with OLP as demonstrated by both the univariate and the multivariate analyses. The clinical features and liver functions of OLP patients with negative or positive HBsAg were nearly the same.

**Conclusions:**

Our findings verify that there is no association between OLP and hepatitis and there is no need to run a screening test for HCV or HBV in OLP patients in China.

**Key words:**Oral lichen planus, hepatitis C virus, hepatitis B virus.

## Introduction

Oral Lichen planus (OLP) is a chronic inflammatory mucocutaneous disorder, mainly affecting the middle aged female patients. It is estimated to occur in 0.2-2.3% of the general population and it represents about 0.6% of all diseases that the dentists frequently meet (1). The clinical characteristics of OLP are white striae which often differentiate into reticular, papular, plaque like, bullous, atrophic, and erosive types. Oral lichenoid lesion (OLL), a disease entity that resembles OLP clinically and pathologically, can be caused by dental materials, drugs or chronic graft versus host diseases (cGvHD). In some instances, OLP and OLL are indistinguishable, only that OLL has a definitive causative agent. Patients with both disease entities can experience no symptoms or nonspecific symptoms of burning, itching, or painful sensations. The characteristics of persistence and liability for malignant transformation remarkably damage the patients’ quality of life.

HCV, a single-stranded ribonucleic acid (RNA) virus, was first identified in 1989 (2). Approximately 40-74% of HCV infected patients have extrahepatic manifestations (3), such as mixed cryoglobulinemia, Non-Hodgkin’s lymphoma, porphyria cutanea tarda, lichen planus, sicca syndrome or thyroid dysfunctions. It was first reported to be associated with OLP in 1991 (4) and since then many researches demonstrate a positive relationship between OLP and HCV infection and even some recommend the screening tests for HCV in OLP patients (5-7). OLP in certain populations can be used as a marker of HCV infection in asymptomatic patients, thus helping the diagnosis, early treatment, and possibly a better prognosis. However, if this is not a true association, the routine testing of OLP patients for HCV may result in the unnecessary use of medical resources, with increased costs and other harmful effects such as the increased anxiety among those tested. So it is very important to identify the association between these two disease categories.

Hepatitis B virus (HBV) is a type of hepadnavirus. Few researches have been done to investigate the association between OLP and HBV infection (6,8-10). Only one study confirmed a weak association between OLP and hepatitis B surface antigen (HBsAg) (9). China has one of the highest HBV carriers prevalence in the world (11). Still little has been known about the relationship between OLP and HBV infection.

Therefore, in this study, we aimed to investigate the relationship between OLP and HCV and HBV infection in China.

## Material and Methods

- Subjects 

Approval from the biomedical institutional review board of Peking University School of Stomatology was received before starting the study (PKUSSIRB-201413033). Data were extracted from the patients’ records in Peking University School of Stomatology from January 2011 to August 2014. The serums of all the participants were collected by the Department of Clinical Laboratory of Peking University School of Stomatology. HCV antibody (HCVAb), HBsAg and liver functions were then measured.

The prevalence of hepatitis C in China is estimated to be 1% (12), and we intended to detect a six-fold increase in the prevalence of hepatitis C among OLP patients with a significance level of 5% (13). The power of the test was set at 80% (β=0.2). As OLP is not a very common disease, we chose a ratio of 2:1 between controls and cases. Based on these assumptions and the equation for estimating sample size, the final sample size of OLP patients was 150, in consideration of 10% loss in follow up rate.

In the current study, one experimental group and three different control groups were enrolled. In total, 776 patients were included. Of which, there were 150 consecutive patients with OLP (Group OLP, the experimental group), 429 inpatients from the Trauma Ward of Oral and Maxillofacial Surgery Department (Group A, the first control group), 110 patients with other oral mucosal diseases, but without a reported association with HCV infection (Group B, the second control group) and 87 patients with oral lichenoid lesion (Group OLL, the third control group). All the patients of Group OLP, Group B and Group OLL were outpatients from the Department of Oral Medicine, Peking University School of Stomatology.

The diagnostic criteria for OLP and OLL were based on the clinical and pathological diagnosis (14,15). Patients in Group A were all the inpatients with maxillofacial trauma from January 2011 to August 2014 and aged over 18 years. Patients in Group B were outpatients with other mucosal diseases including, but not limited to burning mouth syndrome, recurrent aphthous ulcer, chronic cheilitis and geographic glossitis.

Demographic data and information concerning the clinical features of OLP, such as the chief complaint, course of the disease, clinical type of the lesions, sites of involvement and skin lesion, were obtained through the clinical records of all participants.

- Laboratory tests

All the blood samples were collected after an overnight fasting period. HBsAg and HCVAb were detected by an automated chemiluminescence immunoassay (CLIA) analyzer (the Architect i2000 system). Liver functions, such as serum level of alanine aminotransferase (ALT), aspartate aminotransferase (AST), total protein (TP), albumin (Alb), alkaline phosphatase (ALP), and lactate dehydrogenate (LDH), were assayed by an automatic biochemistry analyzer (H7180, Hitachi). Hemoglobin concentration (Hb) and platelet count (PLT) were measured by the automated hematology analyzer (XS-1000i, Sysmex). To confirm the authenticity and reliability, all the data were stored as electrical information by two different researchers.

- Statistical analysis

The collected data were analyzed using SPSS-15.0 software. The differences of the positive rates of HCVAb and HBsAg between the experimental group and the control groups were evaluated by the χ2 test and Fisher exact test (HCVAb between Group OLP and Group B). Parameters of the liver functions were presented as the mean and standard deviation (SD), the differences of which between experimental group and control groups were compared using the independent t test. Then the parameters of the liver functions were recorded into binary variables, which were normal and abnormal (coded as 0 and 1), according to the normal references range. The latter were used for the later statistical analysis. The binary logistic regression analysis was used to measure the association between OLP status and the presence of HCVAb and / or HBsAg as well as the liver functions. All statistical tests were 2-sided, and a value of *P*≤0.05 was considered to be statistically significant.

## Results

The demographic data of the study groups were depicted in  Table 1. There were significant differences regarding the ages between Group OLP and the other three groups. Furthermore, there were more male patients in Group OLP than in Group OLL (*P*=0.01).

**Table 1:**
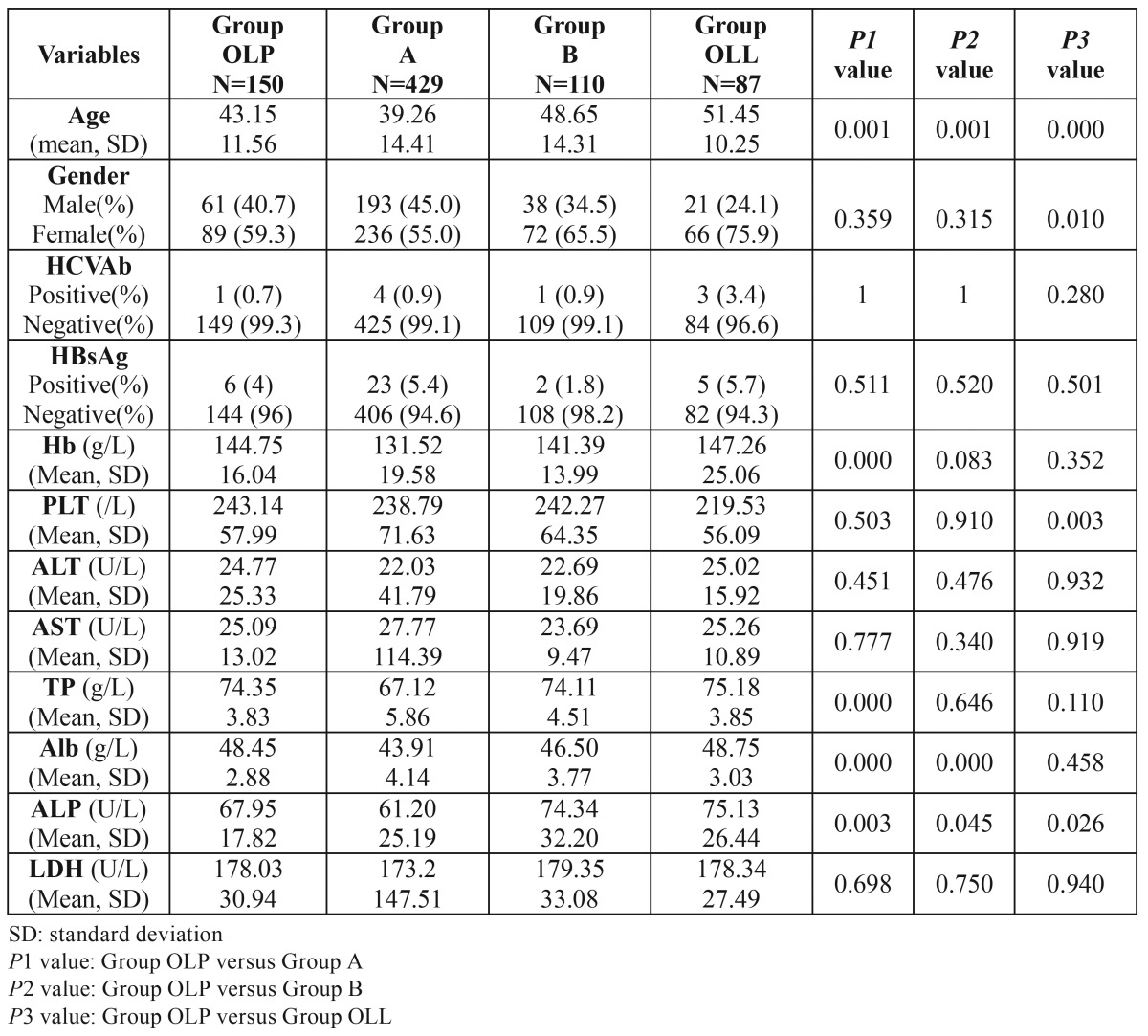
The differences between Group OLP and control groups by univariate analysis.

- Clinical characteristics of OLP patients

**Figure 1:**
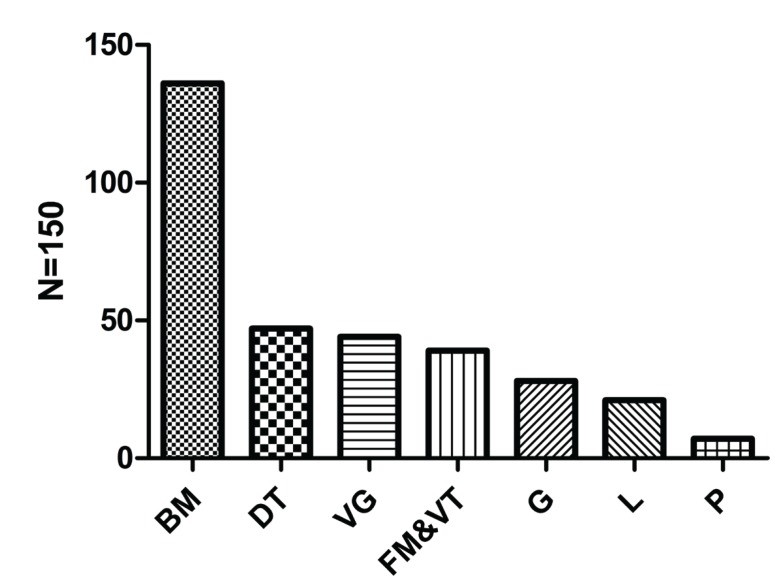
Sites involved in OLP patients. The sites that involved in OLP patients in decending orders. BM, buccal mucosa; DT, dorsum of tongue; VG, vestibular groove; FM&VT, floor of mouth and ventral tongue; G, gingiva; L, lip; *P*, palate.

Half of the 150 OLP patients came to our department presenting with white lesions (82,54.7%) and the rest complained of an irritating pain (37, 24.7%), roughness (4, 2.7%), and other sufferings (22, 14.7%). The shortest duration of the disease was one week while the longest was 20 years with the mean duration of (15.37±34.57) months. The clinical types of the lesions included reticular (61, 40.7%), atrophic (46, 30.7%) and erosive (43, 28.7%) varieties. The most commonly involved sites (as shown in figure 1) were the buccal mucosa (136, 90.7%), the dorsal tongue (47, 31.3%), the vestibular groove (44, 29.3%), the floor of the mouth and ventral tongue (39, 26%), the gingiva (28, 18.7%), the lip (21, 14%), and the palate (2, 1.3%) in a descending order with 12 patients involved with the complexion.

- The association between OLP and HCVAb, HBsAg and liver functions

The positive rates of HCVAb were highest in Group OLL (3/87, 3.4%), identical in Group A and Group B (4/429, 0.9% and 1/110, 0.9%) and lowest in Group OLP (1/150, 0.7%). But none of these differences were significant ( Table 1). The positive rates of HBsAg in Group OLL, Group A, Group OLP and Group B were 5.7%, 5.4%, 4% and 1.8%, respectively. However, the differences among these groups were not significant ( Table 1). The parameters of liver functions were presented as mean±SD ( Table 1). All the values were within normal range. However, Hb, TP, Alb, and ALP in Group OLP were higher than in Group A. There was one inpatient, positive for HBsAg, with dramatical high values of ALT (778U/L), AST (2383U/L) and LDH (3103U/L) in Group A, so the SD of these three parameters was remarkably high. If the evaluation was made without including these data, the results were the same except that the SD of ALT, AST and LDH in Group A would decrease a lot.

In order to exclude the confounding effects of age, gender and other unknown factors, we utilized the binary logistic regression to adjust the multiple factors that were associated with OLP. In all the regression models, the ALT1 (the recoded binary variable of ALT), HCVAb and HBsAg were entered. And the other factors, added in the model, were those with significant differences in the univariate analysis ( Table 2). After adjusting the multiple factors, the binary logistic regression showed there was no association between OLP and HCVAb or HBsAg ( Table 2), the same as the univariate analysis demonstrated above ( Table 1). Although we found that Hb and TP were higher in Group OLP than in Group A ( Table 1), both the mean values were within normal range. When compared to Group B, only the age was found significantly different. The OLP patients were male and younger when compared to OLL patients. And among the 87 patients, none had skin lesions, whereas the 12 OLP patients had a skin involvement. Interestingly, the OLL patients had the highest positive rates of HCVAb and HBsAg. However, the differences were not significant by both the univariate and multivariate analyses.

**Table 2:**
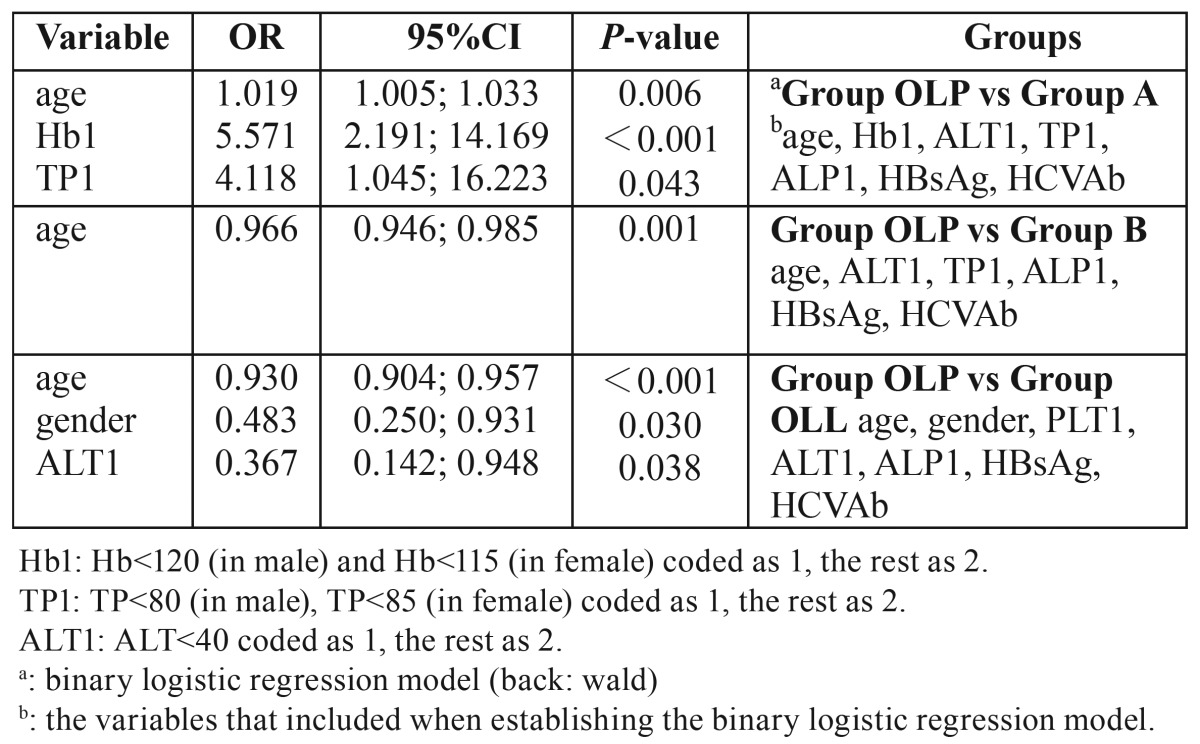
Binary logistic regression for the association between OLP and parameters of liver function, HBsAg, and HCVAb.

- Clinical features of OLP associated with hepatitis

All the four patients with white both clinical lesions and positive HCVAb (1 with OLP and 3 with OLL) were females and aged over 50 years. Two patients suffered from an irritating pain while the other two complained of white lesions. The course of the disease varied from one month to sixty months. All the lesions were bilateral without skin involvement. As there was only one OLP patient with positive HCVAb, it was not possible to compare the differences in regard to the clinical features between positive and negative HCVAb in Group OLP.

Five male and one female OLP were patients positive for HBsAg ( Table 3). Their mean age was (46.67 ±9.09) years (range, 35-54 years). Half patients (n=3) complained of white striae lesions and the remaining suffered from an irritating pain. The lesion types included reticular (n=2), atrophic (n=2) and erosive (n=2) types. The course of the disease varied from 1 month to 12 months with a mean duration of (5.5±5.1) months. There were no differences between HBsAg negative and HBsAg positive OLP patients in relation to the clinical characteristics (age, gender, chief complaints, course of the disease, type of lesions and skin lesions) and the parameters of liver functions by both univariate ( Table 3) and multivariate analyses, except for the serum level of albumin.

**Table 3:**
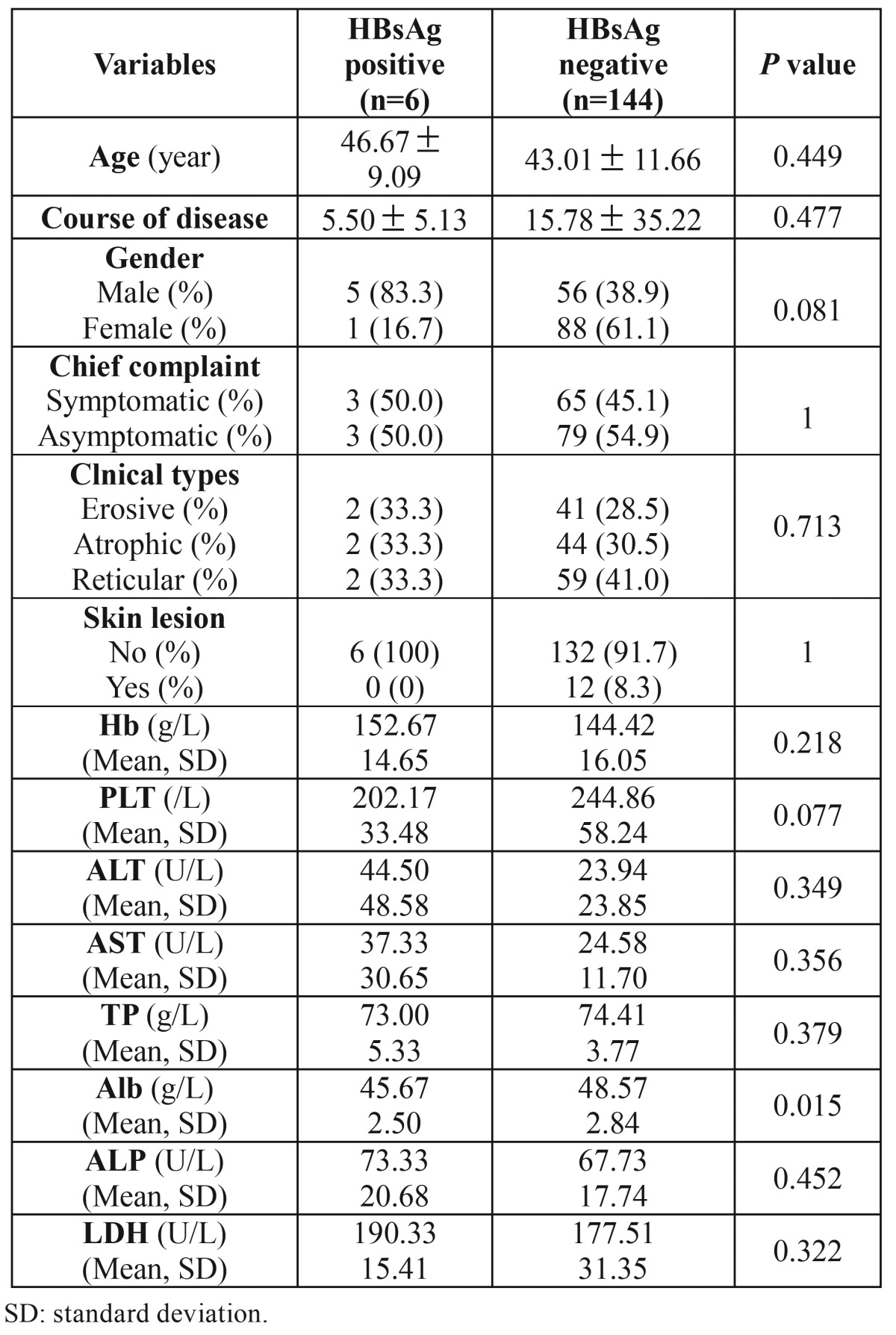
The differences between positive and negative HBsAg patients in Group OLP.

## Discussion

The probable relationship between OLP and liver disease has been discovered since 1978 (16). After HCV was indentified in 1989, several studies have found the coexistence of HCV and OLP. Then in 1991 it was first reported to be associated with OLP (4). A large scale study showed that the prevalence of HCV in OLP patients was three times higher than that of the control group (1.9% and 0.4%, respectively, *P*<0.001) and recommended a screening test of HCV in OLP patients (6). In 2004, Lodi *et al*. carried out a multi-center study to investigate the association between OLP and HCV infection (13) and found that the positive rate of HCVAb in OLP group was seven times as high as that of the control group. In their systemic review, which included 24 case-control studies, the overall odds ratio (OR) was nearly five (OLP group compared to the control group) and the results were different when compared different age groups, control groups (17), different countries or regions and different study designs (13,18).

Despite the enormous papers published in the literature, the relationship between HCV and OLP is still highly controversial. Pilli *et al*. found HCV-specific T-cell responses at the site of the lesions of HCV associated OLP, strongly suggesting a role for HCV-specific T-cell responses in the pathogenesis of OLP associated with HCV infection (19). Whereas, Femiano *et al*. did not find the viral genome in oral epithelium of the patients with both OLP and HCV (20). And still it is very important to find out the true relationship between OLP and HCV. If there is no association between these two diseases, we could avoid the additional psychological pressure and the unnecessary medical resources spent on OLP patients.

In China, few researches (21) have been done in the recent 10 years which demonstrated no relationship between these two disorders. However, in that study, the control group was confusing and needed to be improved. Therefore, in our study we chose three control groups from the same hospital of OLP patients. The estimated HCV prevalence in Chinese population was 1% (12), almost identical to those of the two control groups (Group A and Group B, both were 0.9%). Moreover, these two control groups enabled us to identify the prevalence of chronic HCV/HBV infection in the population referred to the same hospital (22). Although these four study groups were not completely matched as regard age and gender, the binary logistic regression was used to adjust the multi-factors that associated with OLP. The strict inclusion and exclusion criteria were used to select the OLP patients. And there were large proportions of OLL patients among the clinical white striae patients. Accidentally, the positive rate of HCVAb was found to be highest in Group OLL. The main reason might be that the mean age of Group OLL was highest among the study groups (as shown in  Table 1) and the positive rate of HCVAb increases with age (23) as dose the prevalence of OLP. Another reason may be that the histopathological results of the oral white striae lesions associated with HCV infection were more likely to be lichenoid reaction (24). But more researches are still needed to verify this possibility. The seroprevalence of HCVAb was not associated with OLP as testified by both the univariate analysis and the binary logistic regression models. Reviewing the literatures for the relationship between OLP and HCV in the recent 10 years, only three case-control studies showed positive relationship between these two disorders ( Table 4). And the criteria of these studies were not clear. The other seven studies revealed no association between OLP and HCV regardless of the prevalence of HCV in the general populations. Compared to our research, many other reports showed OLP might not be relevant to HCV in certain regions. Therefore, there is no need to run a screening test of HCV in OLP patients in China.

**Table 4:**
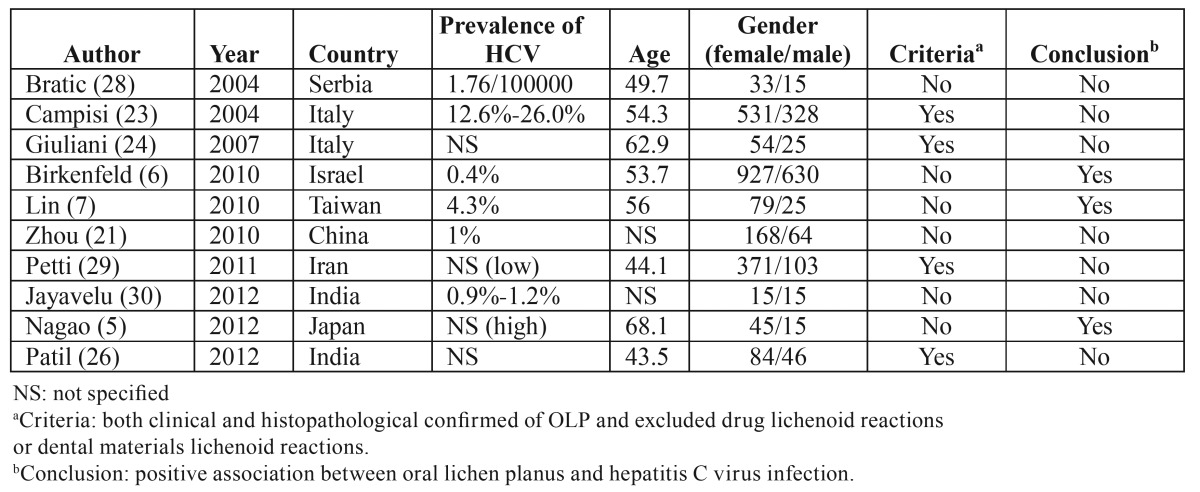
Case-control studies in regard to the association of OLP and HCV in recent 10 years.

Several studies investigated the association between OLP and HBV infection and demonstrated no relationship between these two diseases (6,8). Whereas, in 1990, another study found a weak association between HBV infection and OLP (9) when the association between OLP and HCV infection was not discovered and the author suggested that there might be an indirect relation between non-A non-B hepatitis and LP. In the current study, the positive rates of HBsAg in Group OLL, Group OLP and Group A were higher than in Group B. But none of these differences were significant. So the epidemiological investigation established no relationship between OLP and HBsAg.

The relationship between the parameters of liver functions and OLP varies in few studies. ALT (9,25) and AST (8,9,25) were reported to be higher in OLP patients. In addition, TP, Alb and ALP showed no differences (25). Patil *et al*. found no differences of ALT, AST and ALP between OLP patients and the controls (26). In the current study, the multivariate analysis demonstrated that Hb and TP were found to be higher when compared to Group A. ALT was lower than that of Group OLL. AST, Alb, ALP and LDH showed no differences between Group OLP and the three control groups. And all these values were within normal range. Together with the results of HCVAb and HBsAg, there is no association of hepatitis with OLP, evaluated by the epidemiological study.

The reported main clinical type of OLP patients, most commonly associated with HCV infection, was the atrophic-erosive type (8,9), and the lesions mainly involved the buccal mucosa (8). Whereas, in our study, the OLP patients with HCV infection presented with reticular lesions, which is in concordance to what Romero observed (27). Few researches investigated the clinical features of OLP patients with positive HBsAg. While the present study demonstrated that they were almost identical to those of OLP patients with negative HBsAg.

In conclusion, OLP might not be an extra-hepatic manifestation of HCV infection in Chinese patients. There is no need to run the screening test of HCVAb and HBsAg for OLP patients in China and further fundamental studies are needed to shed the light on the pathogenesis of OLP involved with HCV.
